# Annotations

**Published:** 1898-11-26

**Authors:** 


### ANNOTATIONS.

### Operations on Lunatics.

However much people may choose to say against the lunacy laws in this country there can be no doubt about the large degree of protection that they afford to the lunatic when once he or she has been received into an asylum. Not only does this protection extend to the prevention of the more obvious forms of abuse, but it even covers the question of treatment. Although we would be the last to suggest that medical men should be in any way fettered in the course of treatment they may advise to any patient, it is impossible not to see that the relations between patient and medical man are somewhat peculiar in asylums where to advise is practically to command. Hence, without suggesting a word against the medical superintendents of our great asylums, we cannot but feel that the constant supervision which is exercised by the Commissioners in Lunacy over every detail of asylum work is a great and even a necessary safeguard. For, after all, medical treatment is often a matter of opinion. We do not consult as to matters of certainty, and the very fact that medical consultations are held shows how much medical treatment is a matter of opinion and of judgment. That being so, it is well that in this country the insane should be protected as they are from the faddist; for we must admit that such may exist even in our own ranks. What, for example, are we to think of a paper on "Surgery among the Insane in Canada," which appears in the *American Journal of Insanity*, in which it is stated that on an examination being made of the female lunatics in a certain asylum, it was found that, of 132 patients, there was organic disease of the generative organs in 122, only 10 being free! and that, as a result, 109 out of these 122 cases had been operated on!! Surely this is gynæcology run mad, and one ought to be glad that lunatics in England have the Commissioners in Lunacy between them and such proceedings.

### Sentimental Sanitation.

It is curious to note how, for lack of knowledge, people treat sanitary matters from a sentimental and æsthetic point of view. In nothing does this show itself more than in regard to the disposal of sewage. Undoubtedly it is, on the face of it, a very nasty idea that a vegetable garden should be manured with liquid sewage. But then, from the town point of view, many things in the country are nasty. The old story of the town-bred boy who, after a visit to the country, eschewed milk for evermore is to the point. Dear boy, he had always associated milk with cheery milkmen and shiny tins. But when he went on a visit to the country and saw his grandfather go into a dirty shed and "squeeze it out of an old cow," as in disgusted tones he afterwards described the process to his mother, he was disillusioned and gave it up! To the townsman, who looks upon manure as abomination, no doubt the farmer's reverence for "muck" is something too disgusting for words, yet it is upon the due utilisation of such materials that the whole success of agriculture depends. These remarks are prompted by a perusal of some recent numbers of the *Rangoon Times*, various writers in which have worked themselves into a fine froth and fury at the very idea of the Chinese, who cultivate the vegetable gardens within the cantonment, making use of "liquid excreta" from the barracks and the hospital as manure. That a nuisance may be caused by the particular method adopted for conveying this or any other sort of manure, or by the particular mode in which it is applied, no one will deny but that the "filthy practice" of disposing of such liquid by at once applying it to the land is in any way of necessity a danger to health is a proposition to which we do not think that any sanitarian of experience will assent for a moment. A Chinaman, careless of æsthetics, or perhaps differently-minded about them, carries the stuff in a bucket and digs it straight into his bit of land. We, on the other hand, are swayed by sentiment; we like, at least, to appear clean; so we mix the stuff up with many times its bulk of water, and pour it into more or less leaky drains, and let it wander mile after mile rotting under ground till it reaches the suburbs, when, if the Local Government Board has its way, we at last do just what the Chinaman did at first hand—we put it on the land, and with it we grow vegetables or grass, or whatever we can make to grow by help of such diluted stuff. No doubt by this process the dwellers in the town are saved some annoyance to the senses, but the end is the same, and we but do by aid of many drain-pipes and other appliances what the Chinaman does with a simple pail.

### Typhus Fever.

The occasional occurrence of sporadic cases of typhus fever should serve to remind us that this disease, which used to be so terrible a scourge in London, is not yet dead in our midst. The facts of the most recent case are described in a special report on the subject by Dr. Waldo. A young man of 19, a brush maker, resident in the parish of St. George-the-Martyr, was admitted to Guy's Hospital, where he developed what was recognised to be an attack of typhus fever. When notified of the occurrence, Dr. Waldo (the medical officer of health) at once took steps for the thorough disinfection of the patient's dwelling. As is always the case, there was overcrowding. The house consisted of two rooms, inhabited by a family of six persons—father, mother, and four children, of ages ranging from 19 to 10 years, who all slept in a single upstairs bedroom. The recently-established reception house for the isolation of persons who have been exposed to infection here showed its utility, the remaining five members of the family being lodged there for upwards of fourteen days while the process of disinfection of the rooms was carried out. Thus once again, it is hoped, an epidemic has been prevented. So rare has typhus fever become in London, at least in its recognised form, that only one death from that cause was registered within the metropolitan area during the whole of last year. There can, however, be no doubt that it is always more or less with us. As in diphtheria, it is only when the disease attains a certain virulence that it is recognised, but it seems almost certain that between the widely-scattered cases, as they appear in the notification returns, there is a series of cases which are not recognised, and for which medical men and health officers must always be on the watch. The little outbreak which occurred in Kensington this spring showed how easy it is to overlook the less typical cases by which epidemics are originated.

